# Triflumizole Induces Developmental Toxicity, Liver Damage, Oxidative Stress, Heat Shock Response, Inflammation, and Lipid Synthesis in Zebrafish

**DOI:** 10.3390/toxics10110698

**Published:** 2022-11-17

**Authors:** Lina Bai, Peng Shi, Kun Jia, Hua Yin, Jilin Xu, Xiaojun Yan, Kai Liao

**Affiliations:** 1 School of Marine Sciences, Ningbo University, Ningbo 315211, China; 2 Ningbo No. 2 Hospital, Ningbo 315010, China

**Keywords:** triflumizole, zebrafish, oxidative stress, heat shock response, inflammation, lipid synthesis

## Abstract

Triflumizole (TFZ) toxicity must be investigated in the aquatic environment to understand the potential risks to aquatic species. Accordingly, the adverse effects of TFZ exposure in zebrafish were investigated. Results demonstrate that, after TFZ exposure, the lethal concentration 50 (LC_50_) in 3 d post-fertilization (dpf) embryos and 6 dpf larvae were 4.872 and 2.580 mg/L, respectively. The development (including pericardium edema, yolk sac retention, and liver degeneration) was apparently affected in 3 dpf embryos. Furthermore, the alanine aminotransferase (ALT) activity, superoxide dismutase (SOD) activity, catalase (CAT) activity, and malondialdehyde (MDA) content in 6 dpf larvae were significantly increased. Additionally, the expression of heat shock response genes (including *hsp70*, *grp78*, *hsp90*, and *grp94*), inflammatory genes (including *p65-nfκb*, *il-1β*, and *cox2a*), and lipid synthetic genes (including *srebp1*, *fas*, *acc*, and *ppar-γ*) in 3 dpf embryos was significantly increased, which was also partially observed in the intestinal cell line form *Pampus argenteus*. Taken together, TFZ could affect the development of zebrafish, accompanied by disturbances of oxidative stress, heat shock response, inflammation, and lipid synthesis. Our findings provide an original insight into the potential risks of TFZ to the aquatic ecosystem.

## 1. Introduction

The widespread use of pesticides may endanger aquatic species by contaminating water through surface runoff or leachingOerke [1,2,3]. Triflumizole (TFZ, C_15_H_15_ClF_3_N_3_O), a triazole fungicide (Nippon Soda Co., Ltd., Tokyo, Japan), has not been approved by the European Commission. It is mostly used to prevent powdery mildew and rust in cereals, vegetables, fruit trees, and other crops [4,5,6,7]. It has the potential to hinder the biological synthesis of ergosterol by limiting C_14_-demethylation in sterol [8]. As recently as 2009, 56,231 pounds of TFZ were spilled in California, although the actual level of human exposure is unknown [9]. Further, TFZ has been found in pears, apples, and cucumbers [10]. Environmental risk assessments of TFZ to non-target species must be carried out immediately [11].

Several studies have demonstrated the toxicity of TFZ to aquatic species, such as freshwater algae [12] and fish [13]. Xi et al. (2019) have indicated that TFZ is potentially toxic to freshwater algae in hydrophytic ecosystems [12]. Ecological studies have suggested that TFZ be classified as showing medium toxicity to fish compared to other triazoles [5]. It has also been found that the 72-h lethal concentration 50 (LC_50_) of TFZ for rare minnow (*Gobiocypris rarus*) embryos was 7.11 mg/L, and that it induced abnormal development, extensively modified enzyme activities, and genes expression [10]. Therefore, further toxicological investigation of TFZ for aquatic organisms is required.

Many aquatic species can survive in polluted environments because of defense mechanisms such as antioxidant system and stress response [14]. In fish, toxicant bioaccumulation initiates redox reactions that generate reactive oxygen species (ROS) changing metabolism [15]. Organisms scavenge it using antioxidants including superoxide dismutase (SOD), catalase (CAT), and malondialdehyde (MDA), which are used to assess environmental risk [16]. Furthermore, ROS production is deeply involved in inflammatory reactions [17]. ROS might be accumulated or released due to the increase in oxygen uptake caused by the aggregation of immune cells in damaged tissues. Furthermore, it has been documented that oxidative stress leads to cellular proteins being damaged so that they must subsequently be refolded [18]. Heat shock proteins (HSPs) are the most effectively protective mechanism, and their syntheses increase remarkably with stress. HSPs enable cells to accommodate various xenobiotic factors and naturally derived cytotoxic factors, and previous research has demonstrated the crucial role of HSPs in oxidative stress [19,20].

Some studies have reported that triazoles may affect lipid metabolism, harm embryonic development, and alter the expression of lipid synthetic genes [21,22,23]. In addition to these target mechanisms, TFZ could also be recognized as an obesogen in mice by acting on the peroxisome proliferator-activated receptor-gamma (PPAR-γ) pathway to increase weight [9], this suggests that TFZ could participate in the lipid synthesis of organisms.

Zebrafish have been commonly used to assess the toxicity of pesticides in the environment [24,25]. Our research aim to study the toxicity and lipid synthesis effects of TFZ by exposing 3 d post-fertilization (dpf) zebrafish embryos and 6 dpf zebrafish larvae. Our results will be conducive to an original understanding of the harmful impacts of TFZ on fish and its mechanism.

## 2. Materials and Methods

### 2.1. Chemicals

Triflumizole (Aladdin, China, CAS: 99387-89-0, 99% purity) was dissolved in dimethyl sulfoxide (DMSO) to 40 mg/mL as a stock solution, then diluted in 1 × EM medium (20 × EM medium: NaCl, 17.5 g; KCl, 0.75 g; CaCl_2_ anhydrous, 2.18 g; KH_2_PO_4_, 0.41 g; Na_2_HPO_4_ anhydrous, 0.142 g; MgSO_4_-7H_2_O, 4.9 g) to the exposure concentrations ensuring that the final DMSO volume was less than 0.1%.

### 2.2. Zebrafish Maintenance and Breeding

All zebrafish research procedures were approved by the Ethical Committee of Ningbo University. The AB strain adult zebrafish (*Danio rerio*) (Shanghai FishBio Co., Ltd., Shanghai, China) were maintained in a light/dark cycle of 14/10 h. The zebrafish were fed with freshly hatched brine shrimp twice daily. Water quality was monitored every day to ensure pH 7.3–7.4, conductivity within 450–500 μS, and a temperature of 28 ± 1 °C. Male and female (2:2) zebrafish were placed in isolation in the mating tanks in order to trigger spawning when the next morning lights on. After two hours, the normal embryos were collected and cultivated in 1 × EM medium at 28 °C.

### 2.3. Zebrafish Exposure

**Figure 1:**
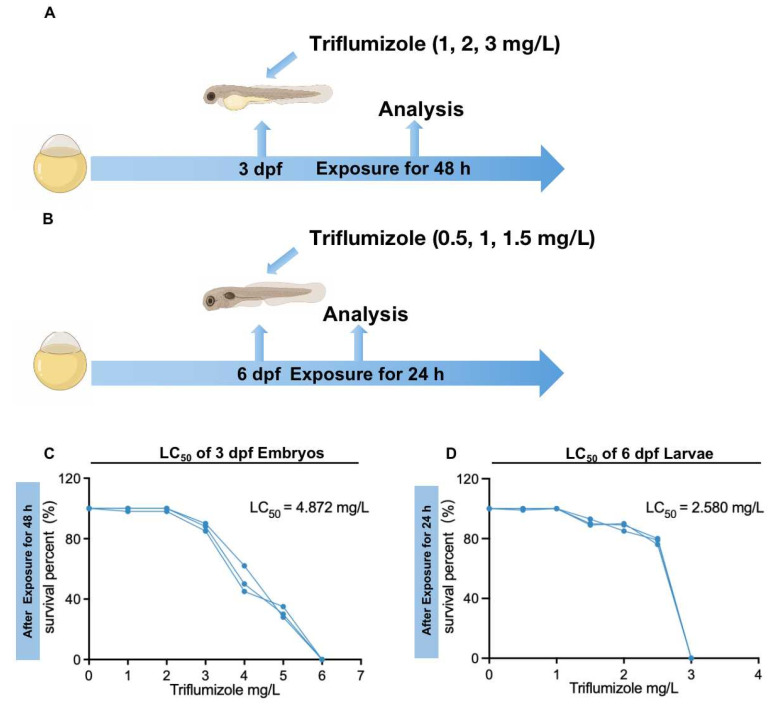
Exposure patterns and the survival percentage of zebrafish after triflumizole exposure. (**A**) Three dpf embryos were exposed to 1, 2, and 3 mg/L triflumizole for 48 h, (**B**) Six dpf larvae were exposed to 0.5, 1, and 1.5 mg/L triflumizole for 24 h. (**C**) The percentage of survival of 3 dpf embryos after triflumizole exposure for 48 h. (**D**) The percentage of survival of 6 dpf larvae after triflumizole exposure for 24 h. dpf: d post-fertilization; LC_50_: lethal concentration 50.

An overview of the exposure procedures is shown in Figure 1A,B. In the control groups, we added 0.1% DMSO in 5 mL 1 × EM medium. We chose 3 dpf zebrafish embryos and 6 dpf zebrafish larvae for TFZ exposure. Firstly, they were exposed to TFZ ranging from 0 to 150 mg/L for 24, 48 and 72 h. the TFZ exposure solutions were replaced per 24 h. Based on the morphological changes and mortality data, 3 dpf zebrafish embryos were exposed to 0, 1, 2, and 3 mg/L TFZ for 48 h. Likewise, 6 dpf zebrafish larvae were exposed to 0, 0.5, 1, and 1.5 mg/L TFZ for 24 h. These were individually dispensed into the 6-well plates, thirty embryos/larvae per well with 5 mL exposure solution, and biological triplicate was collected for each exposure. In order to retain the suit concentrations and water quality, the TFZ exposure solutions were exchanged daily. The 6-well plates were incubated at 28 °C for light/dark cycles of 14/10 h during the exposure period. The survival rates of embryos and larvae were counted in each exposed group every 24 h.

### 2.4. Fish Intestinal Cell Line Exposure

The intestinal cell line obtained from a marine fish, silver pomfret (*Pampus argenteus*), was obtained and cultured as our previous method [26]. Cells were plated on 96-well plates, then exposed to 0, 5, 10, 15, 20, 25, 30, 35, and 40 mg/L TFZ in triplicate for 24 h. We used a Cell Counting Kit-8 (Dojindo, Kumamoto, Japan) to assess cell viability. Briefly, cells were incubated with CCK-8 solution for 4 h at 28 °C. Finally, samples were detected in the absorbance at 450 nm by a microplate reader (BioTek, Winooski, VT, USA). Based on cell viability data, the intestinal cell line was exposed to 0, 5, 10, and 20 mg/L TFZ for 24 h to evaluate its toxicity. Then cells were trypsinized at 37 °C for 1 min and collected for the next analysis.

### 2.5. Morphological Assay

After TFZ exposure, phenotypic changes were evaluated in 3 dpf zebrafish embryos and 6 dpf zebrafish larvae. After anesthetizing with 0.16% tricaine (Sangon Biotech, Shanghai, China), they were fixed on the culture dish in a lateral view using 3% methylcellulose. A lateral view of the entire zebrafish larvae was observed and photographed with a stereomicroscope (Olympus, Tokyo, Japan).

### 2.6. Enzyme Activities Assay

Thirty embryos/larvae (each replicate) were homogenized with 300 μL saline solution, followed by centrifuging at 2500 rpm for 15 min at 4 °C to collect the supernatant for use in subsequent experiments. Three replicates were performed. The activities of SOD, CAT, alanine aminotransferase (ALT), and the content of MDA were assayed by commercially available biochemical assay kits (Nanjing Jiancheng Bioengineering Institute, Nanjing, China) in accordance with the manufacturer’s instructions. The SOD activity was tested by the xanthine oxidase method. In brief, superoxide anions can oxidize hydroxylamine to nitrite and then turn amaranth purple in the presence of a chromogenic agent. Data were recorded by reading the absorbance at 550 nm and computing the activity of the SOD. One unit of SOD activity (U) was defined as the amount of enzyme required to inhibit the oxidation reaction by 50% and was expressed as U/g protein. The CAT activity was tested by the ammonium molybdate method. The decomposition of hydrogen peroxide is quickly terminated by adding ammonium molybdate. The rest of the hydrogen peroxide reacts with ammonium molybdate to form a pale-yellow complex compound, which was detected at 405 nm and used to compute the CAT activity. One unit of CAT activity was defined as the amount of enzyme required to consume 1 μmol H_2_O_2_ in 1 min at 25 °C and was expressed as μmol/min/g protein. The products of lipid peroxidation (measured MDA content) were tested by the thiobarbituric acid (TBA) method, and the amount of TBA substance that occurred through lipid peroxidation was detected after incubation at 95 °C with TBA. The pink color generated in these reactions was detected by spectrophotometry at 532 nm, and MDA content was expressed as nmol/g protein. The pyruvate reacts with 2-4-dinitrophenylhydrazine to generate the hydrazone, which acquires maximum staining by the addition of NaOH to measure ALT activity [27]. It was examined at 505 nm and was expressed as μmol/min/g protein. Protein concentration was tested using the bicinchoninic acid (BCA) protein assay kit (Nanjing Jiancheng Bioengineering Institute, Nanjing, China) for Cu^2+^ following protein-mediated reduction of Cu^2+^ by an alkaline environment. All measurements were performed on a microplate reader, using the A590 microwell plate protocol.

### 2.7. Quantitative Real Time PCR (qPCR)

The total RNA was extracted using Trizol reagent (Omgea, Norcross, GA, USA) according to the manufacturer’s instructions. Next, the samples of RNA were reverse transcribed to generate cDNA using the Reverse Transcription Kit (TRAN, Beijing, China). Each 25 μL application contained 4 μL diluted cDNA, 10 μL qPCR PowerUp SYBR Green Master Mix (TRAN, Beijing, China), 1 μL forward and reverse primers (10 μM), and 4 μL RNA-free water. Two tests were performed for each gene in each biological replicate. The qPCR condition was as follows: 94 °C for 30 s (1 cycle), 94 °C for 5 s, 60 °C for 15 s, and 72 °C for 10 s (45 cycles). The results were subjected to relative quantitative analysis using *β-actin* as an endogenous control gene [26]. The primers used for qPCR are listed in the Table S1. The expression of relative genes was calculated by the 2^–ΔΔCT^ method [18].

### 2.8. Statistical Analysis

SPSS version 20.0 (SPSS Inc., Chicago, IL, USA) was used for statistical analysis. LC_50_ values were determined using GraphPad Prism software version 9.0 (GraphPad Software, San Diego, CA, USA). The data were expressed as mean ± standard error of means (SEM). The results were demonstrated through three independent experiments. One-way analysis of variance (ANOVA) with Tukey’s multiple range test was used to analyze cell activity differences. Differences among two groups were analyzed by unpaired *t*-test with Welch’s correction. * *p* < 0.05 was considered significant, and ** *p* < 0.01 was considered highly significant.

## 3. Results

### 3.1. Exposure Concentrations

To determine the TFZ dose which caused harmful effects in zebrafish, different concentrations were used for exposure and survival rates were recorded. For the primary screening of tolerance ranges in 3 dpf zebrafish embryos and 6 dpf zebrafish larvae, we used 0, 30, 60, 90, 120, and 150 mg/L TFZ. Next, we exposed 3 dpf zebrafish embryos and 6 dpf zebrafish larvae to 0, 1, 2, 3 mg/L and 0, 0.5, 1, 1.5 mg/L TFZ, respectively (Figure 1A,B). After TFZ exposure, the 48 h LC_50_ of 3 dpf zebrafish embryos is 4.872 mg/L (Figure 1C), and the 24 h LC_50_ of 6 dpf zebrafish larvae is 2.580 mg/L (Figure 1D).

### 3.2. Zebrafish Larvae Morphology after TFZ Exposure

**Figure 2:**
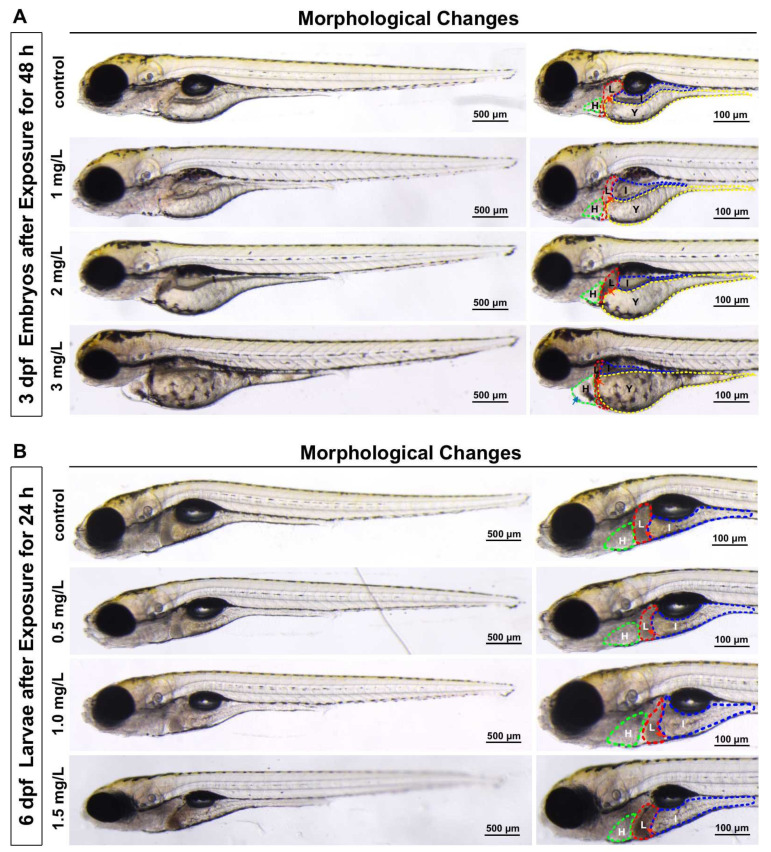
Effects of development after triflumizole exposure in zebrafish. (**A**) 3 dpf zebrafish embryos with morphological changes after triflumizole exposure for 48 h. (**B**) 6 dpf zebrafish larvae with liver degeneration after triflumizole exposure for 24 h. Blue arrows and red arrows indicate pericardial edema and liver degeneration, respectively. H: heart, the green dotted line; L: liver, the red dotted line; I: intestine, the blue dotted line; Y: yolk sac, the yellow dotted line. Scale bars = 500 μm; Scale bars = 100 μm.

To investigate the developmental toxicity of TFZ, morphological changes were scored. After TFZ exposure for 48 h in 3 dpf zebrafish embryos, it was discovered that TFZ induced pericardium edema in the 3 mg/L group, and induced yolk sac retention and liver degeneration in the 2 and 3 mg/L groups (Figure 2A). After TFZ exposure for 24 h in 6 dpf zebrafish larvae, liver degeneration was also noted in 1 and 1.5 mg/L groups, pericardial edema was not observed in 0.5, 1, and 1.5 mg/L groups (Figure 2B).

### 3.3. Enzyme Activities after TFZ Exposure

**Figure 3:**
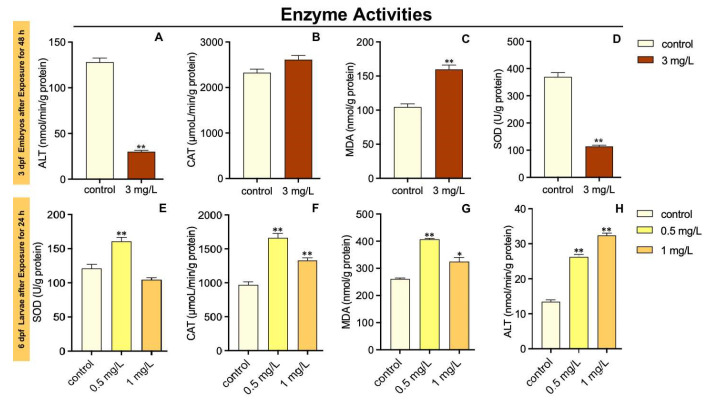
Effects of triflumizole on enzyme activities in zebrafish. For 3 dpf zebrafish embryos, SOD activity (**A**), CAT activity (**B**), MDA content (**C**), and ALT activity (**D**) after triflumizole exposure for 48 h. For 6 dpf zebrafish larvae, SOD activity (**E**), CAT activity (**F**), MDA content (**G**), and ALT activity (**H**) after triflumizole exposure for 24 h. Data are expressed as the mean of three replicates ± standard error (SEM). Asterisks denote significant differences between the control group and TFZ groups (determined by Dunnett post hoc comparison, * *p* < 0.05, ** *p* < 0.01). SOD: superoxide dismutase; CAT: catalase; MDA: malondialdehyde; ALT: alanine transaminase.

The SOD activity in 3 dpf zebrafish embryos was markedly decreased in the 3 mg/L TFZ group relative to the control group (*p* < 0.01, Figure 3A). Compared to the control group, the CAT activity in 3 dpf zebrafish embryos was of no significance in 3 mg/L TFZ group (*p* > 0.05, Figure 3B). Meanwhile, the MDA content in 3 dpf zebrafish embryos was significantly higher in 3 mg/L TFZ group than the control group (*p* < 0.01, Figure 3C). The ALT activity in 3 dpf zebrafish embryos was significantly decreased in 3 mg/L TFZ group compared to the control group (*p* < 0.01, Figure 3D).

The SOD activity in 6 dpf zebrafish larvae was dramatically increased in 0.5 mg/L TFZ group relative to the control group (*p* < 0.01, Figure 3E). However, compared to the control group, the SOD activity in 6 dpf zebrafish larvae was of no significance after 1 mg/L TFZ exposure (*p* > 0.05, Figure 3E). The CAT activity in 6 dpf zebrafish larvae was significantly increased in 0.5 and 1 mg/L TFZ groups compared with the control group (*p* < 0.01, Figure 3F). Meanwhile, the MDA content in 6 dpf larvae was significantly higher in 0.5 (*p* < 0.01) and 1 mg/L (*p* < 0.05) TFZ groups than the control group (Figure 3G). The ALT activity in 6 dpf zebrafish larvae was significantly increased in 0.5 and 1 mg/L TFZ groups compared to the control group (*p* < 0.01, Figure 3H).

### 3.4. Heat Shock Response in Zebrafish Larvae after TFZ Exposure

**Figure 4:**
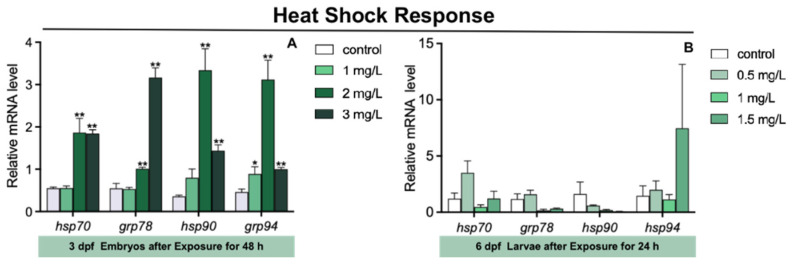
The expression of heat shock response genes (including *hsp70*, *grp78*, *hsp90*, and *grp94*) in zebrafish. (**A**) For 3 dpf zebrafish embryos, the expression of *hsp70*, *grp78*, *hsp90*, and *grp94* after triflumizole exposure for 48 h. (**B**) For 6 dpf zebrafish larvae, the expression of *hsp70*, *grp78*, *hsp90*, and *grp94* after triflumizole exposure for 48 h. Data are expressed as the mean of three replicates ± standard error (SEM). Asterisks denote significant differences between the control group and TFZ groups (determined by Dunnett post hoc comparison, * *p* < 0.05, ** *p* < 0.01). *hsp70*: heat shock protein 70; *grp78*: glucose-regulated protein 78; *hsp90*: heat shock protein 90; *grp94*: glucose-regulated protein 94.

The expression of glucose-regulated protein 94 (*grp94*) in 3 dpf zebrafish embryos was markedly increased in 1 mg/L TFZ group relative to the control group (*p* < 0.05, Figure 4A). Furthermore, compared to the control group, the expression of heat shock protein 70 (*hsp70*), *grp78*, *hsp90*, and *grp94* in 3 dpf zebrafish embryos was significantly increased in 2 and 3 mg/L TFZ groups (*p* < 0.01, Figure 4A).

Compared to the control group, the expression of *hsp70*, *grp78*, *hsp90*, and *grp94* in 6 dpf zebrafish larvae was of no significance in 0.5, 1, and 1.5 mg/L TFZ groups (*p* > 0.05, Figure 4B).

### 3.5. Inflammatory Genes Expression in Zebrafish Larvae after TFZ Exposure

**Figure 5:**
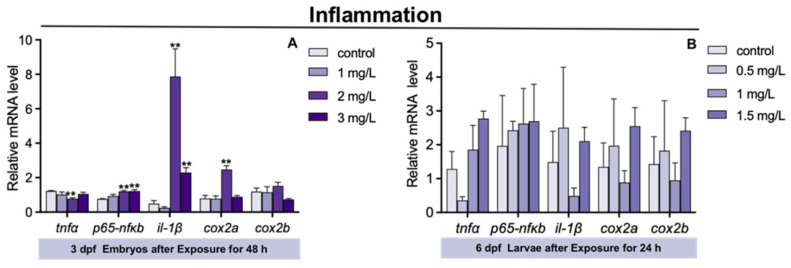
The expression of inflammatory genes (including *tnfα*, *il-1β*, *p65-nfκb*, *cox2a*, and *cox2b*) in zebrafish. (**A**) For 3 dpf zebrafish embryos, the expression of *tnfα*, *il-1β*, *p65-nfκb*, *cox2a*, and *cox2b* after triflumizole exposure for 48 h. (**B**) For 6 dpf zebrafish larvae, the expression of *tnfα*, *il-1β*, *p65-nfκb*, *cox2a*, and *cox2b* after triflumizole exposure for 24 h. Data are expressed as the mean of three replicates ± standard error (SEM). Asterisks denote significant differences between the control group and TFZ groups (determined by Dunnett post hoc comparison, ** *p* < 0.01). *tnfα*: tumor necrosis factor α; *il-1β*: interleukin 1, beta; *p65-nfκb*: p65-nuclear transcription factor κB; *cox2a*: cyclooxygenase type 2 a; *cox2b*: cyclooxygenase type 2 b.

The expression of tumor necrosis factor α (*tnfα*) in 3 dpf zebrafish embryos was significantly decreased in 2 mg/L TFZ group compared to the control group (*p* < 0.01, Figure 5A). Compared to the control group, the expression of p65-nuclear transcription factor κB (*p65-nfκb*), interleukin 1, beta (*il-1β*), and cyclooxygenase type 2 a (*cox2a*) in 3 dpf zebrafish embryos was significantly increased in 2 mg/L TFZ group (*p* < 0.01, Figure 5A). The expression of *p65-nfκb* and *il-1β* in 3 dpf zebrafish embryos was obviously increased in 3 mg/L TFZ group compared to the control group (*p* < 0.01, Figure 5A).

Compared to the control group, the expression of *tnfα*, *p65-nfκb*, *il-1β*, *cox2a*, and *cox2b* in 6 dpf zebrafish larvae was of no significance in the 0.5, 1, and 1.5 mg/L TFZ groups (*p* > 0.05, Figure 5B).

### 3.6. Lipid Synthesis Gene Expression in Zebrafish Larvae after TFZ Exposure

**Figure 6:**
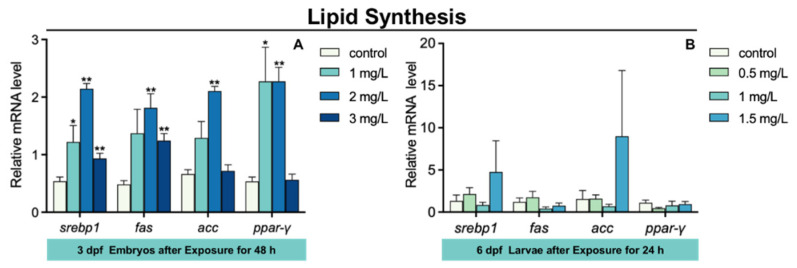
The expression of lipid synthesis genes (including *srebp1*, *fas*, *acc*, and *ppar-γ*) in zebrafish. (**A**) For 3 dpf zebrafish embryos, the expression of *srebp1*, *fas*, *acc*, and *ppar-γ* after triflumizole exposure for 48 h. (**B**) For 6 dpf zebrafish larvae, the expression of *srebp1*, *fas*, *acc*, and *ppar-γ* after triflumizole exposure for 48 h. Data are expressed as the mean of three replicates ± standard error (SEM). Asterisks denote significant differences between the control group and TFZ groups (determined by Dunnett post hoc comparison, * *p* < 0.05, ** *p* < 0.01). *srebp1*: sterol regulatory element binding transcription protein 1; *fas*: fas cell surface death receptor; *acc*: acetyl-coa carboxylase; *ppar-γ*: peroxisome proliferator-activated receptor gamma.

The expression of sterol regulatory element binding transcription protein 1 (*srebp1*) and *ppar-γ* in 3 dpf zebrafish embryos was markedly increased in 1 mg/L TFZ group relative to the control group (*p* < 0.05, Figure 6A). Compared to the control group, the expression of *srebp1*, fas cell surface death receptor (*fas*), acetyl-coa carboxylase (*acc*), and *ppar-γ* in 3 dpf zebrafish embryos was significantly increased in 2 mg/L TFZ group (*p* < 0.01, Figure 6A). The expression of *srebp1* and *fas* in 3 dpf zebrafish embryos was dramatically increased in 3 mg/L TFZ group compared to the control group (*p* < 0.01, Figure 6A).

Compared to the control group, the expression of *srebp1*, *fas*, *acc*, and *ppar-γ* in 6 dpf zebrafish larvae was of no significance in the 0.5, 1, and 1.5 mg/L TFZ groups (*p* > 0.05, Figure 6B).

### 3.7. Effects of TFZ on Fish Intestinal Cell Line

**Figure 7:**
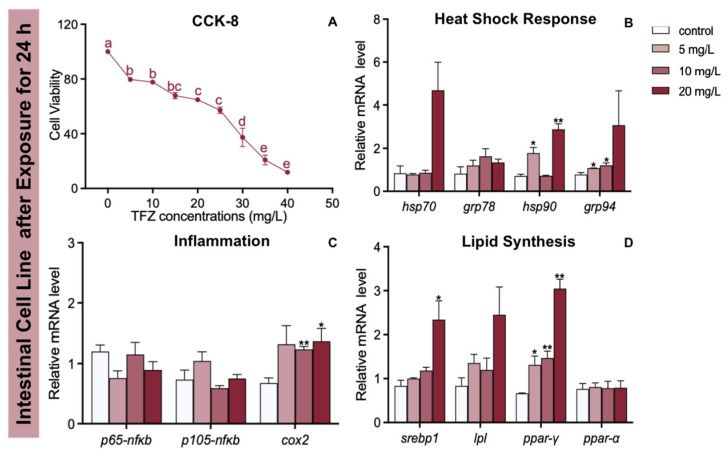
Effects of the fish intestinal cell line after triflumizole exposure for 24 h. (**A**) The cell viability. (**B**) The expression of *hsp70*, *grp78*, *hsp90*, and *grp94* after triflumizole exposure. (**C**) The expression of *p65-nfκb*, *p105-nfκb*, and *cox2* after triflumizole exposure. (**D**) The expression of *srebp1*, *lpl*, *ppar-γ* and *ppar-α* after triflumizole exposure. Data are expressed as the mean of three replicates ± standard error (SEM). Values without a common superscript letter differ (*p* < 0.05, Tukey’s test). Asterisks denote significant differences between the control group and TFZ groups (determined by Dunnett post hoc comparison, * *p* < 0.05, ** *p* < 0.01). Data are expressed as the mean of three replicates ± standard error (SEM). *p65-nfκb*: p65-nuclear transcription factor κB; *p105-nfκb*: p105-nuclear transcription factor κB; *cox2*: cyclooxygenase 2; *lpl*: lipoprotein lipase; *ppar-α*: peroxisome proliferator-activated receptor alpha.

As the TFZ concentration increased in the intestinal cell line, the cell activity generally decreased (Figure 7A). The expression of *hsp90* and *grp94* was increased in the 5 mg/L TFZ group relative to the control group (*p* < 0.05, Figure 7B). Additionally, the expression of *grp94* was significantly increased in the 10 mg/L TFZ group compared to the control group (*p* < 0.05, Figure 7B). Compared to the control group, the expression of *hsp90* was markedly increased in the 20 mg/L TFZ group (*p* < 0.01, Figure 7B). Compared to the control group, the expression of *cox2* was obviously increased in the 10 (*p* < 0.01) and 20 mg/L (*p* < 0.05) TFZ groups (Figure 7C). The expression of *srebp1* was significantly increased in the 20 mg/L TFZ group compared to the control group (*p* < 0.05, Figure 7D). The expression of *ppar-γ* was significantly increased in the 5 (*p* < 0.05), 10 (*p* < 0.01), and 20 mg/L (*p* < 0.01) TFZ groups relative to the control group (Figure 7D).

## 4. Discussion

From our results, it is clear that TFZ strongly affected the early development of zebrafish in a concentration-dependent manner. After TFZ exposure, the 48 h LC_50_ in 3 dpf zebrafish embryos and the 24 h LC_50_ in 6 dpf zebrafish larvae were 4.872 mg/L and 2.580 mg/L, respectively. Previous studies have suggested that the 72 h LC_50_ of TFZ for rare minnow (*Goboicypris rarus*) embryos was 7.11 (6.69–7.51) mg/L [13], which was to some degree higher than the results in this study. The species tested and exposure time caused these differences in acute toxicity experiments. For *Oncorhynchus mykiss*, the acute 96 h LC_50_ was 0.57 mg/L in Lewis’s study [28], Hermsen’s study found that the benchmark concertation of six triazoles ranges from 1.5 mg/L to 25 mg/L in zebrafish embryos [29], which was in accordance with our research.

In addition, we discovered obvious morphological changes induced by TFZ, including pericardial edema, yolk sac swelling, liver size reduction and liver color darkening in 3 dpf zebrafish embryos after exposure for 48 h. Zebrafish embryonic toxicity tests found that pericardial edema and yolk sac retention were extensively noted after triazoles exposure [29,30,31,32]. According to Jiang’s study, difenoconazole can induce liver degeneration, including the retention of yolk sac in zebrafish [33]. It has been reported that a delay in yolk sac uptake is indicative of dysfunction of the liver during zebrafish larval development following tamoxifen exposure [34]. We found that yolk sac retention and liver degeneration occur in a concentration-dependent manner in zebrafish larvae, which was in accordance with the results obtained from a previous report [35]. It is noteworthy that the yolk sac is the only nutrient source in zebrafish larvae during embryonic development, and the retention of the yolk sac may affect nutrient absorption and lipid metabolism [36]. We subsequently observed that ALT activity was obviously increased in the 6 dpf zebrafish larvae after TFZ exposure. In conclusion, morphological changes in the liver and a significant increase in ALT activity indicate liver damage.

The antioxidant defense systems were affected by oxidative stress following TFZ exposure. SOD and CAT are potent enzymes for defending against oxygen toxicity because of their inhibition of effects on oxyradical formation [37]. In addition, the MDA content may indirectly represent the extent of lipid peroxidation. According to our study, SOD activity, CAT activity, and MDA content were significantly higher in 6 dpf zebrafish larvae. The increase in SOD activity, CAT activity, and MDA content is likely to toxicant stress and counteract the damage from ROS [38]. However, SOD activity was strongly inhibited in the 3 mg/L TFZ group in 3 dpf zebrafish embryos, indicating that it might destroy the protective system of zebrafish larvae. We observed that the expression of HSPs (including *hsp70*, *grp78*, *hsp90*, and *grp94*) in 3 dpf zebrafish embryos sharply increased in the 2 mg/L TFZ group, indicating a protective effect against protein misfolding [18]. Furthermore, we found that the expression of *hsp90* in the intestinal cell line was significantly increased in the 5 and 20 mg/L TFZ groups, which was in accordance with 3 dpf zebrafish embryos.

Several studies in aquatic organisms have demonstrated the potential for environmental pollutants to disrupt inflammatory gene expression. Inflammatory genes expressed in zebrafish larvae could be increased by glyphosate exposure [39]. Tricyclazole, a pesticide, also altered the transcription of inflammatory factors such as *tnfα* after exposure [24]. Tissue damage caused by environmental stimuli could lead to inflammation [40]. Our study suggests that TFZ exposure significantly increased the expression of *p65-nfκb*, *il-1β*, and *cox2a* which could be infer that potential tissue damage was caused by TFZ. The expression of *cox2* was markedly upregulated in the 10 and 20 mg/L TFZ groups in the intestinal cell line, which was in accordance with zebrafish larvae.

The increased oxidative stress, heat shock response, and inflammatory response are closely related to lipid synthesis in zebrafish [31,41]. In our research, we found that the expression of *srebp1*, *fas*, *acc*, and *ppar-γ* in 3 dpf zebrafish embryos was markedly increased after TFZ exposure, which was in accordance with the expression of *srebp1* and *ppar-γ* in the intestinal cell line. In reverse, TFZ caused liver damage and lipid synthesis through oxidative stress, heat shock response, and inflammation. Further studies should focus on the mechanism that TFZ contributes to lipid synthesis.

## 5. Conclusions

In summary, our results show TFZ-induced morphological changes (including pericardium edema, yolk retention, and liver degeneration) as well as death in zebrafish embryos. TFZ exposure could cause oxidative stress in zebrafish larvae. Moreover, the upregulation of *ppar-γ* suggests that the PPAR signaling pathway might participate in the imbalance of lipid metabolism. These findings provide a new insight into the potential mechanisms underlying the lipid metabolism imbalance of TFZ in zebrafish.

## Data Availability

Data are contained within the article or Supplementary Materials.

## Supplementary Materials

The following supporting information can be downloaded at: https://www.mdpi.com/article/10.3390/toxics10110698/s1, Table S1: The sequence of primers for qPCR [42,43,44,45,46,47,48,49,50,51].
