# Supplementary material for: Triflumizole Induces Developmental Toxicity, Liver Damage, Oxidative Stress, Heat Shock Response, Inflammation, and Lipid Synthesis in Zebrafish
Source: Toxics. 2022 Nov 17;10(11):698. doi: 10.3390/toxics10110698 (PMC9699234; doi:10.3390/toxics10110698)
Supplement: Supplementary file 1 [file toxics-10-00698-s001.zip › toxics-2007772-supplementary.pdf]

Table S1: The sequence of primers for qPCR.

Species	Primer	Sequence (5′ → 3′)	Reference
Zebrafish	*hsp70*-F	GGAGAAAGCACTGAGGGACG	[42]
	hsp70-R	CTCTGCCGTTGAAGAAATCC	
	grp78-F	AAGAGGCCGAAGAGAAGGAC	[43]
	grp78-R	AGCAGCAGAGCCTCGAAATA	
	hsp90-F	TCAGTTCATCGGTTACCCAAT	[42]
	hsp90-R	TTTGTCTTTGTCTTCGCCTG	
	grp94-F	ATGGCACCAAGAAGAATGACTT	[43]
	grp94-R	GCACCAGCTTTTTACGGATAAC	
	tnfα-F	GCTGGATCTTCAAAGTCGGGTGTA	[44]
	tnfα-R	TGTGAGTCTCAGCACACTTCCATC	
	p65-nfκb-F	GCTGGATCTTCAAAGTCGGGTGTA	[45]
	p65-nfκb-R	CAGTCTTTTCCCACCAGCTC	
	il-1β-F	CATTTGCAGGCCGTCACA	[46]
	il-1β-R	GGACATGCTGAAGCGCACTT	
	cox2a-F	ACTACCCCTGAGCTTCTCACA	[47]
	cox2a-R	GATGCTGTTGATGATATCCCAGATTG	
	cox2b-F	GGCTCATCCTTATTGGTGAGACTAT	[47]
	cox2b-R	TCGGGATCAAACTTGAGCTTAAAATA	
	srebp1-F	GCTAGTTTTCATGGAATAACGC	[48]
	srebp1-R	CATTGAGAAGGGCAGTGTCG	
	fas-F	TGTTGCGTCTTGACTACACTGC	[49]
	fas-R	ATAAAGCCCCCTTCCCTCG	
	acc-F	ACCCCAACAGAGGGAACA	[49]
	acc-R	AAGAGCGGAAGGAAACCA	
	ppar-γ-F	TCTCCGCTGATATGGTGGAC	[50]
	ppar-γ-R	GTCGATGCCTGATATGCTGC	
	β-actin-F	TGCTGTTTTCCCCTCCATTG	[46]
	β-actin-R	TTCTGTCCCATGCCAACCA	
Intestine cell line of silver pomfret	*hsp70-F*	GGAAGCCCAAGATTCAAGTG	By authors
	hsp70-R	GACTGTGATGACTGCGTTG	
	grp78-F	ATCACAAACGACCAGAACCG	By authors
	grp78-R	CGCCAGCACTGCCATAAAG	
	hsp90-F	GAATCACACGACCACGCGA	By authors
	hsp90-R	TGAGGTCCTTGCCAGTGTC	
	grp94-F	TGCTCCACATCACCGACAC	By authors
	grp94-R	GGAAAGCAGAGTAGAAACCC	
	p65-nfκb-F	GAAATGGCGCCTCAGTGTTG	By authors
	p65-nfκb-R	TGCTGGAGCAAGTTGACGAT	
	p105-nfκb-F	AGAACGTGGCGAAGACTCTG	By authors
	p105-nfκb-R	CCACGCTGAGGTCCATTTCT	

cox2-F	CTTCGCACAGCACTTCACAC	By authors
cox2-R	CTGAGATTCGGGGACGTGAG	
srebp1-F	CAGGACATGGAGTTTGGAGG	By authors
srebp1-R	AGGATGGAGGACTTGGAT	
lpl-F	CCCTGCGTTTTCACCTTGTG	[51]
lpl-R	TGCGAAGGAAGGGGAGTTTG	
ppar-γ-F	TGGAAGGGGAGCAGTTCATT	[51]
ppar-γ-R	CGTCTCCTGTCTGCTGATGT	
ppar-α-F	CCACTACCAACCCCCTTCAC	[51]
ppar-α-R	GGAGCTAAGGACGCTGTTGT	
β-actin-F	ACCCTGAAGTACCCCATCGA	By authors
β-actin-R	GGCCACTCTCAGCTCATTGT	
